# Students and Teachers Benefit from Mindfulness-Based Stress Reduction in a School-Embedded Pilot Study

**DOI:** 10.3389/fpsyg.2016.00590

**Published:** 2016-04-26

**Authors:** Sarah Gouda, Minh T. Luong, Stefan Schmidt, Joachim Bauer

**Affiliations:** ^1^Clinic for Psychosomatic Medicine and Psychotherapy, Medical Faculty, Medical Center, University of FreiburgFreiburg, Germany; ^2^Institute for Transcultural Health Studies, European University ViadrinaFrankfurt (Oder), Germany

**Keywords:** mindfulness, schools, students, teachers, mental health, stress, interpersonal

## Abstract

**Objective:** There is a research gap in studies that evaluate the effectiveness of a school-embedded mindfulness-based intervention for both students and teachers. To address this gap, the present pilot study reviews relevant literature and investigates whether students and teachers who participate in separate Mindfulness-Based Stress Reduction (MBSR) courses show improvements across a variety of psychological variables including areas of mental health and creativity.

**Methods:** The study applied a controlled waitlist design with three measurement points. A total of 29 students (*n* = 15 in the intervention and *n* = 14 in the waitlist group) and 29 teachers (*n* = 14 in the intervention and *n* = 15 in the waitlist group) completed questionnaires before and after the MBSR course. The intervention group was also assessed after a 4-month follow-up period.

**Results:** Relative to the control group, significant improvements in self-reported stress, self-regulation, school-specific self-efficacy and interpersonal problems were found among the students who participated in the MBSR course (*p* < 0.05, Cohens' *d* ranges from 0.62 to 0.68). Medium effect sizes on mindfulness, anxiety and creativity indicate a realistic potential in those areas. By contrast, teachers in the intervention group showed significantly higher self-reported mindfulness levels and reduced interpersonal problems compared to the control group(*p* < 0.05, Cohens' *d* = 0.66 and 0.42, respectively), with medium effect sizes on anxiety and emotion regulation.

**Conclusion:** The present findings contribute to a growing body of studies investigating mindfulness in schools by discussing the similarities and differences in the effects of MBSR on students and teachers as well as stressing the importance of investigating interpersonal effects.

## Introduction

The long forgotten etymology of the word “school” is derived from the ancient Greek *scholé,* which originally referred to a mode of freedom and self-determined activity. Scholé in that sense is a state of being in which individuals feel liberated of pressures of all kind—primarily time pressure and the pressure to perform^1^. Etymology notwithstanding, however, modern schools often constitute a source of stress and a miniature representation of an overbearing society's demands on its prospective, fully functional citizens, instead of providing a space for personal growth, creativity, curiosity and learning. Students are faced with a strong emphasis on academic performance, reflected in an often much too narrow focus on grades, strategic future planning and career-oriented learning. The increased importance of time efficiency is particularly evident in the fundamental education reform *G8* that led to the shortening of the school duration at German Gymnasiums (BE: grammar schools, AE: high school) from 9 to 8 years. Notably, despite this contraction, the total instructional time and academic curriculum remained almost entirely unchanged (Büttner and Thomsen, 2015). Teachers too, are affected by stress and overload. As a consequence, they are often forced to strip down their mandate to a bare minimum at the expense of the arguably more rewarding aspects of their profession: the joy of mentoring and conjoined personal growth, the liberty to explore and unlock potential.

The following literature review illustrates the need for change in the educational field exhibited both by students and teachers. First, the specific characteristics and challenges of these target groups are described. We then propose mindfulness-based approaches as one highly suitable and promising course of action. Following a review of the empirical basis of mindfulness, the overall rationale of the present research study is described, and its methods, results and implications presented.

## Background

### Considering the case of students

Particularly older adolescents experience performance pressure due to the high demands of the graduation phase and the far-reaching consequences of academic achievement that can influence their freedom of choice in subsequent careers and professions or even their chances to secure a job in the first place. This is reflected in the finding that the majority of students repeated one of the final three years before graduation (Huebener and Marcus, 2015). High prevalence of pain, health complaints and stress were reported in a sample of 1260 German 10th and 11th grade students (Milde-Busch et al., 2010). Consistent with these findings, the Shell Youth study (Albert et al., 2010) reported that 24% of the respondents (12–25 years old) perceived daily life at school, at university or during their vocational training as stressful and very straining, 60% experienced medium exposure to stress and 16% displayed a relaxed attitude toward external demands. Students attending a Gymnasium spent 38–44 h per week on school-related activities inside and outside of the school environment which resembles the workload of full-time employment (Böhm-Kasper and Weishaupt, 2002). Therefore, it is plausible that a study relying on focus group discussions with students found four out of 10 dimensions of adolescent stress experience to be related to school stress. The study distinguished between stress resulting from school performance, school attendance, teacher interaction and school/leisure conflict. The other adolescent stress domains involved interpersonal problems with family members and the peer group as well as difficulties associated with romantic relationships, future uncertainties, financial pressure and the responsibilities of emerging adulthood. All 10 stress dimensions were correlated positively with anxiety and depression and negatively with self-esteem. These mental health parameters indicate that the critique leveled at the educational system addresses essential risk factors that should be heeded. Gender analysis revealed higher scores among girls in seven of the 10 stress dimensions (Byrne et al., 2007). Numerous studies on gender differences in mental health have consistently pointed to a greater frequency of internalizing symptoms and disorders for females relative to males. According to a multi-national survey that accumulated data from more than 160,000 subjects (11–15 years old) from 29 European countries as well as Canada, USA and Israel, girls were more likely to report poorer health regardless of the country (Cavallo et al., 2006). Particularly girls in late adolescence were more susceptible to chronic somatic complaints (Barkmann et al., 2010). Self-rated assessments of health-related quality of life dimensions including physical and psychological wellbeing, mood and emotions, and self-perception also revealed lower scores among adolescent girls (Michel et al., 2009). By contrast to these epidemiological studies which used gender as an independent variable, the qualitative study by Wiklund et al. (2010) aimed to understand and contextualize the underlying causes of female distress in light of sociocultural and gender-theoretical perspectives. The authors concluded that the young women's narratives predominantly incorporated modern discourses of striving for productivity, efficiency and effectiveness as well as other explanatory factors that have been attributed to gender differences in mental health. One of these risk factors was discussed in terms of a greater vulnerability to interpersonal stress among females. Regardless of gender aspects, the role of stress experiences in early life and their persistent detrimental effects are of major societal concern, particularly since adolescence is the peak time for the onset of many mental health disorders (Kessler et al., 2005; Paus et al., 2008; Lee et al., 2014). According to a large health survey on psychopathological problems and psychosocial impairment in children and adolescents in Germany, 17.8% of young people aged 14–17 years showed an increased risk for a mental health disorder as assessed by the Strengths and Difficulties Questionnaire (Hölling et al., 2014).

Adolescent risks and vulnerabilities notwithstanding, young people are also provided with opportunities for personal growth, social learning, resilience, and creativity as they become more advanced and flexible in the use of their cognitive control skills (Crone and Dahl, 2012). These cognitive control skills or executive functions involve processes such as attention regulation, impulse inhibition, decision-making, and working memory (Blakemore and Choudhury, 2006). Neuroimaging studies have demonstrated that the performance of executive functions is distinctly paralleled by activations in the prefrontal cortex (e.g., Duncan, 2001; Niendam et al., 2012). Furthermore, research has revealed that the prefrontal cortex undergoes a prolonged maturation process throughout adolescence until the early twenties. In particular, profound structural and functional reorganizations have been found in the prefrontal cortex. Some areas affected by these changes have been labeled the “social brain” due to their role in tasks that require self-reflection as well as perspective taking in order to read the emotions or mental states of others (Blakemore, 2008; see also Waytz and Mitchell, 2011). These capacities are of great importance as adolescents engage in more diverse and complex relationships beyond the family sphere and undergo social role transitions. While adolescence is characterized by growing independence from caretakers, it is also a developmental period of increased sensitivity toward peer admiration and peer rejection. Thus, it is crucial to consider the social and motivational context that has been shown to exert a strong influence on adolescents‘ decisions and behaviors, thereby leading to more positive or more negative trajectories (Crone and Dahl, 2012). Overall, adolescence is regarded as a highly adaptable transitional period that can establish a foundation for future health (Sawyer et al., 2012). Remarkable longitudinal studies that followed the development of individuals from birth throughout the course of their life until their thirties highlighted the importance of emotional health and self-regulation skills in childhood and subsequently as predictors for adult life-satisfaction, health, wealth and public safety. The practical implications of these striking findings were discussed in terms of interventions that promote these key prognostic factors (Moffitt et al., 2011; Layard et al., 2014). From the perspective of developmental neuroscience, it has also been suggested that it may be particularly worthwhile to support the trainable skills of the “social brain” through the school curriculum while the adolescent brain is still being shaped (Blakemore, 2010; Sanger and Dorjee, 2015).

### Considering the case of teachers

There are strong indications that the teaching profession is associated with considerable health risks. According to several wide-scope studies (Schaarschmidt, 2005; Bauer et al., 2006; Bauer, 2009; Hillert et al., 2013), around 20–30% of German teachers are suffering from serious stress-related health issues, including severe burnout. Similarly, in a sample of over 400 German teachers, an investigation into coping styles with respect to work load and conditions indicated that 50.2% of these teachers are at risk for burnout and severe stress (Bauer et al., 2006). Indeed, teaching ranks among the professions highest in burnout rates (Schaarschmidt, 2005). In a large-sample study investigating specific burnout symptoms, Unterbrink et al. (2007) found that German teachers report high levels of emotional exhaustion, depersonalization and low personal achievement, relative to comparable studies in Germany and abroad. The authors postulate a significant imbalance between invested effort and obtained reward (measured by the “Effort-Reward Imbalance Questionnaire,” Siegrist et al., 2004) as a contributing cause due to its association with mental health risks. Effort-reward imbalance and over-commitment have also been linked to reduced immune function in stressful situations in a sample of German teachers (Bellingrath et al., 2010), while immune function, in turn, was associated with teacher burnout (Von Känel et al., 2008). Other studies also point to heavy workloads, adverse conditions and mental health risks among German teachers (Bauer et al., 2006, 2007). Specifically, class size and difficult, hostile behavior on the part of students have been cited as distressing factors shaping the work life of German teachers (Bauer et al., 2006, 2007). Amidst hostile interactions that may mount to actual verbal and physical transgressions (Unterbrink et al., 2008; Bauer, 2009), teachers often lack the opportunity to develop productive and growth-generating relationships to their students or cultivate a creative, trustful climate in their classrooms. This is all the more fatal since it is precisely the relationship to students that constitutes one of the most powerful influences on teacher mental health (Bauer et al., 2007; Unterbrink et al., 2007, 2008; Hattie, 2009; McCormick and Barnett, 2011).

Due to the immense value and potential that teaching holds, this profession is also worthy of continued scientific attention and initiatives beyond health and clinical perspectives, which have been the focus of most investigations in this area. This entails a constantly renewed focus on resources inherent in this occupation. In fact, one might argue that it is precisely some of the frequently reported stressors which teachers encounter that represent the most promising resources of this profession. The interactions with students, parents and colleagues, so often cited as a major source of stress (e.g., Friedman, 2000), may constitute particularly fruitful and personally enjoyable aspects of teachers' daily lives if the right context and circumstances are provided. Admittedly, the confrontation with classrooms can be experienced as draining, the interactions with parents as threatening and the relationship to colleagues as cold or even hostile. However, an environment is also conceivable where those same stressors are perceived positively. Teachers may develop an emotionally enriching connection to students, tap the support and common goals that they may share with parents and obtain a sense of cohesion and inspiration from their colleagues. Not only would such a context maximize resources and satisfaction, it would also prevent more clinical developments that could lead to critical stress levels and burnout. The Job Demands-Resources Model (Demerouti et al., 2001) for instance holds that the interaction of job demands and resources available to the individual eventually leads to burnout symptoms. Similarly, other resource-based stress concepts such as the Conservation of Resources Theory (Hobfoll, 2001) emphasize the many ways in which individuals maintain and foster their resources and prevent burnout. Interestingly, Hobfoll (2001), whose theory exhibits a notable contextual focus, in his list of central cross-cultural resources names several that the school system may inherently enhance, such as “feeling valuable to others,” “positively challenging routine,” “role as a leader,” “sense of commitment,” “companionship,” “feeling that my life has meaning/purpose,” “people I can learn from,” and “providing children's essentials.” In an innovative piece of research, Klusmann et al. (2008) posit that individual self-regulation types may contribute to whether the teaching context is experienced as stressful or rewarding. The authors draw on the four types of coping patterns identified by Schaarschmidt et al. (1999), who conclude that a coping style characterized by both high engagement and resilience is associated with the most favorable mental health parameters. In the same vein, Klusmann et al. (2008) found that a self-regulation pattern that meets these criteria scored lowest on emotional exhaustion and highest on job satisfaction across the four types. Similarly, this pattern of self-regulation was linked to positive ratings of teaching quality and student motivation. Accordingly, research on how to promote teacher self-regulation so as to balance work commitment and stress resilience should be of considerable value to the field of education and teacher health.

Along similar lines, Friedman (2000) describes how teachers who entered their profession with high motivation, commitment and idealism are met with a reality so at odds with their expectations that they are unable to retain their initial enthusiasm and notions of good teaching and become subject to exhaustion and, potentially, burnout. In what he considers a healthy coping style, teachers may learn to adjust their expectations and standards so as to lower the discrepancy between expected and factual professional efficacy. In other words, learning to adapt one's expectation of oneself and of one's performance to more realistic standards may help increase teachers' personal sense of self-efficacy. While it is plausible and indeed empirically validated that self-efficacy is a valuable resource (Bandura, 1977, 1986; Dicke et al., 2014), we consider the dedication that often motivates a career in teaching an invaluable asset to both the individual teacher and the school system at large. Processes whereby teachers are forced to compromise their dedication in an attempt to self-preserve and function often risk squandering this asset. The challenge then is rather to equip teachers with skills and tools that enable them to make full use of the resources available to them. This is in line with a study arguing that teachers' coping strategies and perceptions of their environment can actively shape and modify the teacher-working environment fit which is crucial to their well-being and teaching skills (Pietarinen et al., 2013). The authors indeed report that proactive coping strategies on the part of teachers are negatively associated with burnout symptoms and perceived work-environment fit. This is especially true for a process that they label *co-regulation*, which refers to the identification and activation of social resources.

In sum, much of the existent literature on teachers' health and teaching as a profession points to (a) significant stress and health risks in this career path, and (b) the importance and potential of both individual and interpersonal resources, including self-regulation and interactions with key persons in this domain.

### Mindfulness: definition, operationalization, and empirical findings

In light of the above, how can a more enabling, resource-oriented school be achieved both for teachers and students? While the discourse around society's current values and their impact on our notion of education is vehement and ongoing, many responses have already arisen to engender a more nurturing educational atmosphere. One conglomerate of such responses, broadly labeled Mindfulness-Based Interventions draws on eastern meditation traditions, specifically the practice of mindfulness. Although mindfulness is a term that carries a wide range of connotations and meanings, it is maybe best described as consistently directing one's attention to the present experience in a deliberate and non-judgmental manner (Schmidt, 2011). Stemming from religious and spiritual roots, mindfulness draws on an explicit value system that emphasizes wholesome attitudes including generosity, kindness, equanimity, compassion, and appreciative joy (Grossman, 2015).

Physicians and psychologists have been reaping the benefits of this concept by means of a secular 8-week program titled *Mindfulness-Based Stress Reduction (MBSR*; Kabat-Zinn, 1984). While MBSR is by no means the only application of mindfulness in a modern context, it has enjoyed wide popularity and strong empirical evidence. The classic MBSR course usually consists of eight meetings of two and a half hours in addition to one full day of mindfulness practice, incorporating formal meditation exercises as well as informal practices that aim to transfer attained insights and attitudes into day-to-day life (Kabat-Zinn, 1984). Thus, course participants learn to meditate and observe internal and external stimuli in a variety of contexts. Simultaneously, they are invited to cultivate a mindful approach to all aspects of their lives: thoughts, emotions, behavior, activities, surroundings and relationships. Furthermore, participants are instructed in simple yoga exercises and psycho-educational contents about stress and mental health. Participants are urged to practice on a daily basis to properly root mindfulness in their everyday lives and allow the concept—which is a thoroughly experiential one—to come to full fruition. The underlying notion is strikingly intuitive: human beings who have learned to focus their attention on their present experience, in all its facets, with acceptance, kindness and openness will find their immediate realities richer and more rewarding, and will cope with adversities more effectively.

From early on, MBSR has been used in the fields of medicine and psychology to assist patients suffering from chronic psychological and physiological ailments, however non-clinical populations increasingly benefit from MBSR as well. The clinical effectiveness of MBSR and mindfulness across a number of disorders and symptoms has been repeatedly demonstrated, with evaluation culminating in promising meta-analyses: In a meta-analysis of 39 studies investigating mindfulness-based therapy at large in diverse clinical populations, Hofmann et al. (2010) reported moderate effect sizes on anxiety and mood (Hedges's *g* = 0.59 and 0.63, respectively), with effect sizes increasing in samples restricted to anxiety and mood disorders (Hedges's *g* = 0.97 and 0.95, respectively). Khoury et al. (2013) reported medium effect sizes with regard to mental and physical symptoms (with average effect sizes of *d* = 0.55 for pre-post effects, *d* = 0.52 for waitlist controlled effects and *d* = 0.33 for treatment controlled studies, respectively) across a sample of 209 studies investigating mindfulness-based interventions in clinical and non-clinical populations. Similarly, Eberth and Sedlmeier (2012) in their meta-analysis of the psychological effects of mindfulness meditation reported large effect sizes with respect to stress, well-being and anxiety in non-clinical samples (*d* = 0.78, 0.80, and 0.64, respectively). Notably, the authors described larger effect sizes for MBSR compared to other forms of mindfulness meditation, potentially indicating a conglomerate of mechanisms (as opposed to a more specific effect of “pure” mindfulness) active in MBSR. In a meta-analysis examining the effects of meditation at large across 163 studies (Sedlmeier et al., 2012), effect sizes for mindfulness-based studies were comparable to overall meditation effect sizes when possible publication biases were accounted for. Overall effect sizes in the medium range were reported for interpersonal changes (*r* = 0.44), reduced state anxiety (*r* = 0.37), trait anxiety (*r* = 0.32) and negative emotion (*r* = 0.34). Effect sizes ranging from small to medium were found with respect to learning and memory (*r* = 0.21), negative personality traits (*r* = 0.18) and emotion regulation (*r* = 0.17).

In addition to the growing body of meta-analyses, studies increasingly investigate the biological and physiological effects of mindfulness. For instance, Davidson et al. (2003) demonstrated that following an 8-week mindfulness intervention, the experimental group (*n* = 25) showed higher brain activity (left-sided anterior activation) which is associated with positive affect than the waiting group (*n* = 16). Likewise, the authors reported indicators of higher immune functioning (as per a greater rise in antibody titers in response to a vaccine). Another study found that following an MBSR course, participants (*n* = 17) showed changes in gray matter density in brain areas associated with learning, memory, emotion regulation and perspective taking, relative to a control group (*n* = 17; Hölzel et al., 2011; see also Fox et al., 2014). However, research on neuronal effects and mechanisms of mindfulness is in its early stages and needs further inquiry to elucidate the often methodologically flawed findings (Tang et al., 2015).

Whereas the almost exponential increase in mindfulness studies has contributed to a solid evidence base for the effectiveness of adult mindfulness-based interventions in non-clinical and clinical populations across various contexts, research on mindfulness for children and adolescents is still in its infancy. Therefore, it is not surprising that research in this area has been facing methodological challenges (Felver et al., 2015). Although just a small number of methodologically rigorous studies have been published to date, the few existing systematic reviews indicate that mindfulness-based interventions are promising for children and adolescents (Black et al., 2009; Meiklejohn et al., 2012; Waters et al., 2014; Weare, 2014b; Felver et al., 2015). Black et al. (2009) concluded in their review of treatment efficacy that interventions based on sitting meditation as a core element have proven to be effective in mitigating children‘s and adolescents‘ preexisting physiological (*d* = 0.16–0.29) and psychosocial/behavioral problems (*d* = 0.27–0.70). The meta-analysis by Zenner et al. (2014) included 24 studies (*n* = 1348) and resulted in a small to medium overall effect size (Hedge‘s *g* = 0.40), subsuming the domains of stress (*g* = 0.39), resilience (*g* = 0.36), emotional coping (*g* = 0.19), and third person ratings (*g* = 0.25). By contrast to the meta-analysis of Sedlmeier et al. (2012) on the effects of adult mindfulness-based interventions, mindfulness studies with children and adolescents yielded the largest effect size for the domain of cognitive performance (*g* = 0.80). Another meta-analysis by Zoogman et al. (2014) obtained a small effect size of *d* = 0.23 after inspecting 20 studies (*n* = 1772) that compared a mindfulness-based intervention for children and adolescents with an active control group. Their comparison of non-clinical and clinical samples showed a significantly greater effect size for the latter group (*d* = 0.50 vs. 0.20). It is also noteworthy that children and adolescents who start off with low levels of executive function skills benefit more from mindfulness-based interventions (Flook et al., 2010). Nonetheless, addressing only these target groups in school settings may stigmatize participants. Therefore, in our opinion adopting a universal school program is more appropriate.

### Rationale of the current study

As illustrated above, schools increasingly exert pressure on students and teachers to perform under overly high demands of efficiency and effectiveness. This tendency can negatively affect the mental health of students and teachers, preventing the educational space from being just that: an actual unrestricted *space* for growth-generating education that unlocks the potential to explore and self-actualize. The project at hand hopes to offset the perceived phenomena of time compression and performance pressure by introducing a mindfulness-based intervention to both students and teachers. Mindfulness may provide participants with a useful tool that may help them maneuver the hassles of daily life as well as with insights about themselves, their realities and their choices. For instance, participants may learn to reappraise unhealthy and excessive demands in favor of more adequate self-care. Mindfulness has been shown to be effective in coping with stress and furthering well-being in a variety of different contexts, and has proven of particular usefulness in the educational field. However, research inquiring into the benefits of mindfulness in German schools remains scarce. The lack of studies that implement and evaluate a mindfulness approach that targets both students and teachers constitutes yet another research gap. Such an approach is likely to lead to synergistic effects, contributing to positive interpersonal interactions and to an overall improved school climate (Flook et al., 2013). To our knowledge, there is hitherto no study that examines a dual approach to teacher and student mindfulness training. To address this gap, we offered both students and teachers of one school participation in separate but parallel MBSR courses. Beside the evaluation of the feasibility and effectiveness of a MBSR course in a school context, this pilot study is among the first to shed light on the similarities and differences in the effects of MBSR on student and teacher populations, respectively. Based on the theoretical foundations and empirical evidence of adult mindfulness programs, it is assumed that adolescent mindfulness programs induce analogous processes and effects among younger target groups. However, as suggested by Burke (2014), there may be developmental distinctions in the mechanisms of mindfulness for adolescents and adults. Bearing in mind the different developmental tasks and the different biopsychosocial conditions in adolescence and adulthood, the present study postulates that (i) students and teachers who participate in separate MBSR courses will show improvements in mental health, wellbeing and creativity compared to a waitlist control group, and (ii) that students and teachers will vary in their response to mindfulness practices.

## Methods and materials

The study followed a controlled waitlist design with three measurement time points, taking place across the two terms of the school year. Time points were at the beginning of the first term (t1, baseline), at the end of the first term (t2, post-test) and at the end of the second term (t3, 4-month follow up). Half of all participating students and teachers were assigned to the course in the first school term, while the second half, having functioned as a control group in this period, was offered course participation in the second term. All participants reported quantitative data at all-time points, resulting in a comparison between the intervention and control group, and 4-month follow-up data for the intervention group. In addition, after each course (i.e., the course in the first and second term) all participants were interviewed in depth about their individual experience. Qualitative results will be reported elsewhere.

A power analysis that assumed an average effect size for mindfulness based intervention of approx. *d* = 0.5–0.6 (as per meta-analyses to date) yielded a necessary sample size of approximately 48 participants per population and group to arrive at a power of 1− β = 0.80.Throughout the project's duration, three schools will be recruited; this pilot study recruited the first of three cohorts that together will meet the required sample size.

### Sample

Two separate samples—from the teacher and student population, respectively—were recruited at a Catholic Gymnasium for girls in Freiburg, Germany^2^. The school administration was first approached with an invitation to participate in the project. During a detailed project presentation, the entire teaching staff and the students of grade 11 were then informed about the project's rationale and contents. Recruitment followed a universal approach addressing the entirety of grade 11 as well as the school's teaching staff. Participation took place on a voluntary basis. Informed consent or parental consent in the case of minors was collected from all participants. Participants were assigned to intervention and waitlist group depending on their schedules and time preference. The study was submitted to and approved by the ethical commission of the university medical school in Freiburg.

### Intervention and procedures

The intervention consisted of the standard 8-week Mindfulness–Based Stress Reduction (MBSR) group program (Kabat-Zinn, 1984). In line with the manualized MBSR instructions, the intervention was delivered by a certified mindfulness teacher and trained Psychiatrist with long-standing experience (with no involvement from the research team). More specifically, the course consisted of eight 2-h sessions in addition to one full day of formal and informal mindfulness meditation and yoga exercises held on school premises. Participants were also encouraged to engage in independent daily practice. Because the project is part of an interdisciplinary Collaborative Research Centre that investigates the topic of *schole*^1^, participating students underwent three 90-min-sessions of theoretical input addressing different aspects of *schole'*. Prior to the beginning of the course, students attended an introductory philosophical lecture on *schole'*. Two further sessions halfway through and at the end of the course were incorporated into school lessons. In this setting, philosophical concepts of work and leisure as well as equanimity were discussed

### Measures

A comprehensive self-report test package was administered to participants in a group setting. Students and teachers completed the same set of standardized questionnaires encompassing a variety of psychological outcome variables. All instruments were screened for appropriateness for the educational context, for the respective age groups and for usability in non-clinical samples. The instruments were also either validated across adolescent populations or included norm tables for this study's age groups in the respective manuals, except for ERSQ, FMI, and PSQ. Likewise, all instruments are widely established and demonstrate solid psychometric properties, as reported by the original authors. In cases where psychometric properties were investigated by other researchers, additional references are cited. The test package required approximately 45 min to complete.

#### Self-reported mindfulness

The level of self-reported mindfulness was measured by means of the 14 item short form of the *Freiburg Mindfulness Inventory* (FMI; Walach et al., 2006) which covers a two-dimensional structure with the factors mindful presence and non-judgmental acceptance.

#### Stress

To assess stress, the German version of the *Perceived Stress Questionnaire* (PSQ; Fliege et al., 2001) was used. The PSQ measures subjective stress perception across 20 items and four dimensions (worries, tension, joy, and demands) that target both generic stressors and stress reactions.

#### Anxiety and depression

In order to assess levels of anxiety and depression, the German version of the Hospital Anxiety and Depression Scale (HADS; Hermann-Lingen et al., 2005) was administered. The HADS consists of 14 items comprising an anxiety and a depression subscale. Indicators of validity and reliability of this screening instrument were reported by Bjelland et al. (2002), Jörngården et al. (2006) as well as White et al. (1999) for use with adolescents.

#### Test anxiety

One of the most common stress sources among students, test anxiety, was measured with the *Test Anxiety Inventory* (TAI) by Schwarzer and Jerusalem (1999). Across 10 items, the scale measures two aspects of examination anxiety: agitation/emotionality (5 items), which addresses emotional and physiological components of test anxiety, and worrying (5 items), which includes more cognitive aspects.

#### Self-efficacy

This construct refers to the optimistic beliefs in one‘s competence and coping abilities when one is confronted with difficult situations. Self-efficacy can be measured across different domains or in a specific situation. The *General Self-Efficacy Scale* (SES-G) comprises 10 items. For the educational context, domain-specific self-efficacy scales were developed to assess the *School-Related Self-Efficacy* (SES-S) of students (Schwarzer and Jerusalem, 1999) and *Teacher Self-Efficacy* (SES-T; Schwarzer and Hallum, 2008).

#### Self-regulation

The *Self-Regulation Scale* (SRS; Schwarzer and Jerusalem, 1999) was used to assess participants' attention control in goal pursuit. The 10 items of the self-report scale cover both aspects of attention regulation and emotion regulation that are particularly crucial in situations when individuals face difficulties in maintaining their goal-oriented behavior.

#### Emotion regulation

The *Emotion Regulation Skills Questionnaire* (ERSQ; Berking and Znoj, 2008) was employed to capture changes in emotional competences. Composed of 27 items, the ERSQ primarily explores coping with negative emotions and includes 9 subscales: awareness, sensations, clarity, understanding, modification, acceptance, resilience, self-support, and readiness to confront distressing situations.

#### Interpersonal competences

The German version of the *Inventory of Interpersonal Problems* (IIP-D; Horowitz et al., 2000) is a self-rating questionnaire for assessing relational difficulties. The short version with 64 items reflects 8 dimensions that can be excessively exhibited (domineering, vindictive, cold, socially avoidant, non-assertive, exploitable, overly nurturant, intrusive).

#### Openness

This variable was measured with the *Openness to Experience* Scale of the German Version of the *NEO Five-Factor Inventory* (NEO-FFI; Borkenau and Ostendorf, 1994). The scale consists of 12 items inquiring into personality facets such as curiosity, creativity, and readiness to engage in new or unusual experiences.

#### Creativity

Changes in creativity were measured with the *Test for Creative Thinking- Drawing Production* (TSD-DP; Urban and Jellen, 1995) in order to assess this construct beyond the dimension of verbal skills. The test presents participants with figural fragments that they are required to complete freely. The resulting drawings are scored on 14 categories (e.g., new elements, unconventionality). Evaluators of this test were blinded to group assignment. Bröcher (1989) and Dollinger et al. (2004) discussed validity and reliability of the measure.

#### Work engagement

The student and employee version of the *Utrecht Work Engagement Scale* (Schaufeli et al., 2002) was used to measure the three dimensions of work engagement: vigor (6 items), dedication (5 items) and absorption (6 items). The scale has been applied in the school contexts with students and teaching staff (Schaufeli et al., 2006; Cadime et al., 2016).

### Fidelity of implementation

As per the guidelines provided by Feagans Gould et al. (2015), the four central dimensions of fidelity are adherence, dosage, quality, and responsiveness. The authors define these components as follows: “(1) adherence—the extent to which the core components were implemented as designed; (2) dosage—the amount of the intervention received by participants; (3) quality—the extent to which an instructor delivered program content as intended; and (4) responsiveness—the extent to which participants were engaged in the program” (Feagans Gould et al., 2015, p. 8).

In the study at hand, adherence was ensured prior to the course through the standardized MBSR course manual as well as regular meetings with the course teacher before and throughout the course. Similarly, adherence was assessed retrospectively through qualitative interviews after the intervention with participants and the course teacher as well as a psychometric measure of mindfulness. As reported below, mindfulness scores changed in the hypothesized direction and qualitative interviews indicated that course contents are consistent with standard MBSR components. With respect to dosage, the course teacher kept an attendance list for every course appointment while interviews after the course revealed the extent to which participants practiced independently. Quality was ensured through reliance on a certified MBSR teacher with extensive experience, as was confirmed in participant interviews. Responsiveness was also tackled in the interviews, where information regarding practice frequency, satisfaction and engagement with the course was provided. While individual responsiveness and practice frequencies varied, the detailed results of the qualitative data analysis will be reported elsewhere.

### Data analysis

Quantitative data was analyzed with the *Statistical Package for the Social Sciences 21* (IBM SPSS 21). Comparisons between the intervention group and the control group relied on Analyses of Covariance (ANCOVA), with baseline test scores serving as a covariate. The effect size Cohen's *d* was calculated based on the adjusted group means and the pooled standard deviation. *T*-tests for independent samples and chi-square tests were conducted to investigate group differences at baseline level. A one-way repeated measures Analysis of Variance (ANOVA) with time as the within-subjects factor was conducted to evaluate changes to the outcome variables across t1, t2, and t3 for the intervention group only. Bonferroni corrections were performed for *post-hoc* pairwise comparisons. The corresponding effect size was reported in partial eta squared (η^2^). The evaluation was carried out by the research team with no involvement from the mindfulness teacher.

## Results

### Students

A total of 29 students (*M*_*age*_ = 16.2, *SD* = 0.51) was recruited for the study (15 in the intervention and 14 in the waitlist group). Fifteen students in the intervention group (*M*_age_ = 16.1, *SD* = 0.51) completed the test package at t1, t2, and t3. In the waitlist group (*M*_age_ = 16.4, *SD* = 0.50), 14 students participated in the assessment at t1 and t2. Socio-demographic data including age (*F* = 1.13, *p* = 0.25) and previous experience with meditation (χ^2^ = 1.03, *p* = 0.31) and/or mindfulness (χ^2^ = 0.00, *p* = 0.96) did not vary significantly between the two groups. No significant group differences between intervention and waitlist group were found at baseline for any of the outcome variables. Means and standard deviations of all outcome measures across all three time points for the student intervention group and t1 and t2 for the waitlist group are provided in Table 1.

**Table 1:** **Means and standard deviations across all three time points for the student intervention group (*n* = 15) and t1 and t2 for the student waitlist group (*n* = 14)**.

**Students Variable**	**Group**	**T1 *M* (*SD*)**	**T2 *M* (*SD*)**	**T3 *M* (*SD*)**
FMI	IG	2.67 (0.49)	2.76 (0.43)	2.92 (0.49)
	WL	2.74 (0.26)	2.59 (0.36)	
PSQ	IG	2.29 (0.38)	2.28 (0.48)	2.09 (0.43)
	WL	2.38 (0.39)	2.69 (0.54)	
HADS-A	IG	7.10 (2.82)	6.67 (3.20)	6.53 (3.09)
	WL	8.71 (3.29)	9.57 (3.39)	
HADS-D	IG	4.50 (3.18)	4.67 (3.24)	3.20 (2.86)
	WL	4.92 (3.17)	6.11 (3.68)	
TAI-E	IG	2.29 (0.72)	2.01 (0.86)	1.97 (0.73)
	WL	2.11 (0.58)	2.17 (0.66)	
TAI-W	IG	2.87 (0.65)	2.76 (0.69)	2.69 (0.77)
	WL	3.09 (0.67)	2.99 (0.65)	
SES-S	IG	2.85 (0.54)	3.00 (0.53)	2.86 (0.63)
	WL	2.62 (0.52)	2.53 (0.44)	
SES-G	IG	2.69 (0.51)	2.76 (0.39)	2.81 (0.50)
	WL	2.78 (0.38)	2.65 (0.31)	
SRS	IG	2.80 (0.31)	2.92 (0.40)	3.01 (0.41)
	WL	2.60 (0.52)	2.44 (0.53)	
ERSQ	IG	2.38 (0.47)	2.47 (0.59)	2.59 (0.57)
	WL	2.21 (0.52)	2.10 (0.51)	
IIP	IG	1.37 (0.41)	1.15 (0.35)	1.12 (0.33)
	WL	1.33 (0.30)	1.37 (0.36)	
NEO-O	IG	2.86 (0.45)	2.84 (0.51)	2.74 (0.58)
	WL	2.53 (0.51)	2.46 (0.52)	
TCT-DP	IG	30.07 (10.55)	34.60 (8.04)	31.00 (9.14)
	WL	30.71 (9.19)	30.29 (7.69)	
UWES	IG	3.17 (0.86)	3.15 (0.82)	2.74 (0.96)
	WL	2.58 (0.88)	2.52 (0.57)	

IG, intervention group; WL, waitlist group; FMI, Freiburg Mindfulness Inventory; PSQ, Perceived Stress Questionnaire; HADS-A, Hospital Anxiety and Depression Scale—Anxiety; HADS-D, Hospital Anxiety and Depression Scale—Depression; TAI-E, Test Anxiety Inventory—Emotionality; TAI-W, Test Anxiety Inventory—Worrying; SES-S, Self-Efficacy Scale—School-related; SES-G, Self-Efficacy Scale—General; SRS, Self-Regulation Scale; ERSQ, Emotion Regulation Skills Questionnaire; IIP, Inventory of Interpersonal Problems; NEO-O, NEO Five Factor Inventory—Openness; TCT-DP, Test for Creative Thinking—Drawing Production; UWES, Utrecht Work Engagement Scale.

Group differences and inference statistics are depicted in Table 2. Significant group differences were found for stress, self-regulation, school-related self-efficacy and interpersonal problems (*p* < 0.05, Cohens' *d* ranges from 0.62 to 0.68). For stress, an increase in the waitlist control group and a stable level of perceived stress in the intervention group were found. The intervention group also reported higher levels of mindfulness, emotional competences and general self-efficacy as well as lower levels of anxiety and the emotionality aspect in test anxiety. The waitlist control group, by contrast, deteriorated on these outcome variables (*p* < 0.10, Cohens' *d* ranges from 0.66 to 0.43). Even though not all group comparisons differed significantly, the intervention group showed improved scores on almost all variables in the hypothesized direction. The worrying-subscale of the test anxiety measure showed non-significant decreases in both the intervention and waitlist group.

**Table 2:** **Univariate ANCOVA on post-test scores covarying for pretest scores comparing the student intervention group (*n* = 15) and student waitlist group (*n* = 14) on the outcome measures**.

**Students Variable**	**IG Adjusted mean _T2_**	**WL Adjusted mean _T2_**	***F***	***df***	***P***	***d***
FMI	2.78	2.57	3.703	1, 26	0.065	0.53
PSQ	2.31	2.65	4.340	1, 26	0.047	0.67
HADS-A	7.01	9.20	3.450	1, 26	0.075	0.66
HADS-D	4.82	5.94	1.377	1, 26	0.251	0.32
TAI-E	1.93	2.26	3.279	1, 26	0.082	0.43
TAI-W	2.83	2.91	0.154	1, 26	0.698	0.11
SES-S	2.92	2.62	6.899	1, 26	0.014	0.62
SES-G	2.79	2.62	3.077	1, 26	0.091	0.48
SRS	2.84	2.53	6.384	1, 26	0.018	0.66
ERSQ	2.42	2.16	2.219	1, 26	0.079	0.47
IIP	1.14	1.38	9.273	1, 26	0.005	0.68
NEO-O	2.69	2.62	0.516	1, 26	0.479	0.14
TCT-DP	34.69	30.19	2.643	1, 26	0.116	0.57
UWES	2.97	2.72	2.025	1, 26	0.167	0.35

The results of the repeated measures ANOVA are displayed in **Table 5**. A significant time effect was yielded for self-reported mindfulness, self-regulation, interpersonal problems, and engagement. Pairwise comparisons showed that significant changes on these outcome variables occurred between t1 and t3. Significant changes on IIP also occurred between t1 and t2, while significant changes on UWES occurred between t2 and t3. All other outcome variables remained relatively stable at follow-up.

### Teachers

A total of 29 teachers (*M*_age_ = 45.9, *SD* = 8.52) was recruited for the study (14 in the intervention and 15 in the waitlist group). Fourteen teachers in the intervention group (*M*_age_ = 47. 2, *SD* = 7.26) completed the test package at t1, t2, and t3, while 15 teachers in the waitlist group (*M*_age_ = 43.9, *SD* = 9.22) completed the test package at t1 and t2. Socio-demographic data including sex (χ^2^ = 0.66, *p* = 0.68), age (*F* = 0.76, *p* = 0.29), previous experience with meditation (χ^2^ = 3.13, *p* = 0.07) and/or mindfulness (χ^2^ = 0.68, *p* = 0.41), workload (*F* = 2.30, *p* = 0.96) and work experience (*F* = 0.80, *p* = 0.23) did not vary significantly between the two groups. No significant group differences between intervention and waitlist group were found at baseline for any of the outcome variables. Means and standard deviations of all outcome measures across all three time points for the teacher intervention group and at t1 and t2 for the waitlist group are provided in Table 3.

**Table 3:** **Means and standard deviations of outcome measures across all three times for the teacher intervention group (*n* = 14) and at t1 and t2 for the teacher waitlist group (*n* = 15)**.

**Teachers Variable**	**Group**	**T1 *M* (*SD*)**	**T2 *M* (*SD*)**	**T3 *M* (*SD*)**
FMI	IG	2.57 (0.47)	2.81 (0.45)	2.81 (0.42)
	WL	2.60 (0.34)	2.58 (0.33)	
PSQ	IG	2.39 (0.38)	2.22 (0.39)	2.29 (0.46)
	WL	2.43 (0.38)	2.36 (0.39)	
HADS-A	IG	9.71 (2.89)	7.29 (3.24)	8.00 (2.63)
	WL	8.60 (3.27)	8.07 (2.34)	
HADS-D	IG	5.14 (2.80)	3.93 (2.56)	4.93 (3.17)
	WL	4.67 (2.64)	4.33 (2.50)	
SES-T	IG	2.96 (0.27)	2.98 (0.21)	3.01 (0.21)
	WL	2.87 (0.46)	2.83 (0.40)	
SES-G	IG	2.84 (0.39)	2.89 (0.25)	2.90 (0.15)
	WL	2.73 (0.24)	2.85 (0.22)	
SRS	IG	2.86 (0.40)	2.89 (0.29)	2.79 (0.30)
	WL	2.83 (0.37)	2.85 (0.37)	
ERSQ	IG	2.55 (0.49)	2.86 (0.47)	2.82 (0.48)
	WL	2.89 (0.38)	2.69 (0.47)	
IIP	IG	1.58 (0.37)	1.39 (0.39)	1.45 (0.40)
	WL	1.55 (0.33)	1.53 (0.42)	
NEO-O	IG	3.05 (0.34)	3.17 (0.32)	3.11 (0.30)
	WL	2.99 (0.42)	3.03 (0.31)	
TCT-DP	IG	24.14 (10.64)	24.14 (11.63)	25.43 (10.07)
	WL	29.13 (8.22)	24.00 (8.62)	
UWES	IG	3.81 (0.72)	3.77 (0.55)	3.71 (0.75)
	WL	3.75 (0.91)	3.54 (0.91)	

IG, intervention group; WL, waitlist group; FMI, Freiburg Mindfulness Inventory; PSQ, Perceived Stress Questionnaire; HADS-A, Hospital Anxiety and Depression Scale—Anxiety; HADS-D, Hospital Anxiety and Depression Scale—Depression; SES-T, Self-Efficacy Scale—Teacher; SES-G, Self-Efficacy Scale—General; SRS, Self-Regulation Scale; ERSQ, Emotion Regulation Skills Questionnaire; IIP, Inventory of Interpersonal Problems; NEO-O, NEO Five Factor Inventory—Openness; TCT-DP, Test for Creative Thinking—Drawing Production; UWES, Utrecht Work Engagement Scale.

Group differences and inference statistics are depicted in Table 4. Significant group differences were found for self-reported mindfulness and interpersonal problems (*p* < 0.05, Cohens' *d* = 0.66 and 0.42, respectively). In confirmation of our hypotheses, a significant increase in mindfulness in the intervention group compared to the waitlist group (*p* < 0.05) was revealed. Interpersonal Problems were likewise reduced significantly in the intervention group relative to the waitlist group.

**Table 4:** **Univariate ANCOVA on post-test scores covarying for pretest scores comparing the teacher intervention group and teacher waitlist group on the outcome measures**.

**Variable**	**IG Adjusted mean _T2_**	**WL mean _T2_**	***F***	***df***	***p***	***d***
FMI	2.83	2.57	5.56	1, 26	0.03	0.66
PSQ	2.23	2.35	0.818	1, 26	0.37	0.30
HADS-A	7.01	8.32	2.00	1, 26	0.17	0.46
HADS-D	3.76	4.49	1.17	1, 26	0.29	0.29
SES-T	2.96	2.86	1.46	1, 26	0.24	0.31
SES-G	2.87	2.87	0.002	1, 26	0.98	0.00
SRS	2.90	2.84	0.42	1, 26	0.52	0.18
ERSQ	2.91	2.65	2.59	1, 26	0.12	0.55
IIP	1.37	1.54	4.512	1, 26	0.04	0.42
NEO-O	3.15	3.05	1.50	1, 26	0.23	0.32
TCT-DP	25.02	23.18	0.24	1, 26	0.63	0.18
UWES	3.74	3.56	1.148	1, 26	0.24	0.24

Further in line with our hypotheses, increases were found in the intervention group with respect to teacher-specific self-efficacy and emotion regulation, relative to the waitlist group, however not at a significant level. Non-significant decreases in perceived stress, anxiety, and depression as well as non-significant increases in general self-efficacy, self-regulation and openness were found in both intervention and waitlist group. Creativity remained stable in the intervention group and was non-significantly decreased in the waitlist group. Engagement was slightly reduced in both the intervention and waitlist group, contrary to our hypotheses. Effect sizes across group differences ranged between Cohen's *d* = 0.00 and 0.55.

The results of the repeated measures ANOVA are displayed in Table 5. One way repeated measure ANOVAs of within-group differences in the intervention group across the three time points yielded no significant changes in outcome measures at follow-up, indicating relatively stable outcomes over time.

**Table 5:** **Repeated measures ANOVA for the student intervention group (*n* = 15) and teacher intervention group (*n* = 14) across all three time points**.

**Variable**	**Students**^**a**^				**Teachers**^**b**^			
	***F***	***df***	***p***	***η^2^***	***F***	***df***	***P***	***η^2^***
FMI	7, 18	2, 13	< 0.01	0.525	2.65	2, 12	0.11	0.306
PSQ	7.18	2, 13	0.19	0.225	0.98	2, 12	0.41	0.140
HADS-A	0.13	2, 13	0.88	0.020	3.90	2, 12	0.05	0.394
HADS-D	3.12	2, 13	0.08	0.324	3.23	2, 12	0.08	0.350
TAI-E	2.62	2, 13	0.11	0.287	–	–	–	–
TAI-W	0.29	2, 13	0.76	0.042	–	–	–	–
SES-S/T	2.51	2, 13	0.12	0.278	0.24	2, 12	0.79	0.039
SES-G	0.786	2, 13	0.48	0.108	0.19	2, 12	0.83	0.031
SRS	6.76	2, 13	0.01	0.510	2.66	2, 12	0.11	0.307
ERSQ	1.07	2, 13	0.37	0.141	2.02	2, 12	0.18	0.252
IIP	9.13	2, 13	< 0.01	0.584	4.21	2, 12	0.04	0.413
NEO-O	1.86	2, 13	0.20	0.222	2.55	2, 12	0.12	0.298
TCT-DP	3.11	2, 13	0.08	0.324	0.12	2, 12	0.89	0.020
UWES	8.87	2, 13	< 0.01	0.577	0.24	2, 12	0.79	0.039

a As determined by pairwise comparisons, significant changes on FMI, SRS, IIP, and UWES occurred between t1 and t3. Significant changes on IIP were also yielded between t1 and t2 as well as on UWES between t2 and t3.

b All significant changes occurred between t1 and t2 as determined by pairwise comparisons. There were no significant differences between t1 and t3 for any outcome variable.

## Discussion

The present findings point to significant effects of MBSR among both students and teachers. Affected variables and their implications are discussed in the following for students and teachers, respectively.

### Students

With reference to students, the comparison between the intervention group and the waitlist control group demonstrated that participation in an MBSR-course resulted in significant improvements with respect to (i) perceived stress (*d* = 0.67), (ii) self-regulation (*d* = 0.66), (iii) school-related self-efficacy (*d* = 0.62), and (iv) interpersonal problems (*d* = 0.68). In general, all outcome variables, with the exception of engagement and openness, changed in the hypothesized direction, and may well yield significant results in a larger sample. The observed magnitudes of the effect sizes exceed the effect sizes for psychological variables that were reported in a recent meta-analysis on mindfulness-based interventions in schools (Zenner et al., 2014).

Although research on MBSR-adaptations and implementations for adolescents—especially in German school contexts—is still limited, these results are consistent with international studies to date. A similar pilot study by Broderick and Metz (2009) examined the effectiveness of a mindfulness-based program that was embedded in the regular school curriculum. The intervention group (*n* = 120) also comprising students (aged 16–19 years) from a private Catholic high school for girls reported significant decreases in negative affect as well as significant increases in feelings of calmness, relaxation and self-acceptance, relative to a small control group (*n* = 17). The same author's subsequent study with students in grades 10–12 from a public high school complemented the findings of the pilot trial. Specifically, the authors found significantly lower levels of perceived stress and psychosomatic complaints and significantly higher levels of self-regulation efficacy among the participating students (*n* = 129) compared to the control group (*n* = 87; Metz et al., 2013). In another study by Kuyken et al. (2013), the effectiveness of a school-based mindfulness curriculum for adolescents (aged 12–16) was evaluated in a large non-randomized controlled trial allocating six schools to the intervention arm (*n* = 256) that were matched with equivalent control schools (*n* = 266). Relative to the control group, fewer depressive symptoms, lower levels of perceived stress and higher levels of well-being were found among the students who engaged in mindfulness lessons. Since measurements were also conducted during the most demanding phase of the school year, it was suggested that mindfulness may boost resilience in stressful times.

With respect to the observed beneficial effect on stress in our study, mindfulness may operate as a buffer against the impact of stress experiences over the course of the school year. Since all participating students signed up voluntarily for the MBSR course, it is assumed that their motivation ranged from non-specific interests to specific needs for building up their stress resilience. The preliminary results from our pilot study showed an increase in perceived stress in the waitlist control group relative to the intervention group. Notably, post-measurement of both groups was conducted before the Christmas break when many exams were scheduled as well. The results concerning student stress converge with the so-called “mindfulness stress buffering account” by Creswell and Lindsay (2014) which has been supported by empirical evidence from their recent randomized controlled trial. Here, lower levels of self-reported stress perceptions to the Trier Social Stress Test (TSST) were found in two conditions: (i) after completion of a brief mindfulness training or (ii) in participants with high levels of pre-existing trait mindfulness. By contrast, participants who were allocated to the active control group or who scored low on trait mindfulness reported the highest perceived stress levels during the TSST. In essence, mindfulness has been regarded as a trainable capacity (Davidson and McEwen, 2012) that contributes to stress resilience by strengthening the “top-down” regulatory pathway while simultaneously mitigating the “bottom-up” stress reactivity pathway (Creswell et al., 2014).

Similarly, based on the “iterative information processing model” by Cunningham et al. (2007) it has been suggested that mindfulness practice may foster the development of self-regulation skills due to the activation of neural networks that are responsible for top-down processing. In particular, top-down processes are defined as awareness and reflection processes that allow for (i) a more elaborate consideration of the situation and context factors and (ii) an emotional reappraisal that also involves the skills of psychological disidentification, cognitive flexibility and inhibitory control. At the same time, the model postulates that bottom-up processes in terms of arousal and emotional reactions may be mitigated more quickly through the practice of mindfulness (Zelazo and Lyons, 2012). During a mindfulness exercise, participants are often invited to turn their attention toward their breath or a different anchor that helps them to stay present-focused. As soon as they notice that their attention drifts into mind-wandering or gets attached to certain emotions or physical sensations, they are encouraged to acknowledge the internal events and to bring their attention gently back to the actual focus of the practice. As a result, different aspects of self-regulation covering cognitive, emotional, physiological and behavioral components can be enhanced through the practice of non-judgmental observation of the changes in one‘s mental states and the initiation of intentional self-regulation processes (Teper et al., 2013). Enhancing self-regulation among students is particularly valuable since it has been postulated as a central personality variable impacting the teaching-learning process at large (De la Fuente et al., 2014). Thus, De la Fuente et al. (2015) found that personal self-regulation was associated with “deep learning” (i.e., intrinsically motivated and curiosity-driven learning), self-regulated learning, performance and learning satisfaction among university students.

Whereas the link between mindfulness and self-regulation has often been the focus of investigation, no school-based mindfulness intervention study was found to date that specifically assessed the effects on self-efficacy. However, the great importance of addressing students' self-efficacy beliefs was emphasized in a previous meta-analysis that documented a positive relationship between self-efficacy and academic performance and persistence (Multon et al., 1991). More recent studies have consistently highlighted the major role of self-efficacy as a key process variable influencing students' academic and career development (Zimmerman et al., 1992; Bandura et al., 1996, 2001; Richardson et al., 2012; Zuffianò et al., 2013). In light of the present results, our data suggests that MBSR can be regarded as a promising intervention to support students‘ school-related self-efficacy.

Likewise, there is also a research gap in assessing the effect of school-based mindfulness interventions on the interpersonal level. The decrease in interpersonal problems in the intervention group compared to the waitlist group is of particular relevance since conflicts with adults like parents and teachers as well as peers constitute a major source of stress during adolescence (Byrne et al., 2007). Bearing in mind that our sample consisted exclusively of female students, research suggesting that especially female adolescents exhibit a greater vulnerability to interpersonal distress (e.g., Benenson and Christakos, 2003; Wiklund et al., 2010) becomes relevant. Complementary to the findings of MBSR studies that showed significant reductions in interpersonal problems among adolescents with heterogeneous clinical diagnoses (Biegel et al., 2009; Sibinga et al., 2011), MBSR in the study at hand also demonstrated significant reductions in interpersonal problems among a non-clinical population of female adolescents. Whether MBSR shows comparable effects on male adolescents has yet to be investigated.

Regarding the findings at 4-month follow-up, a possible delayed effect on mindfulness was found. Even though no significant effect was revealed at t2, the intervention group seems to have benefited from the course over time, resulting in significant changes at t3, compared to t1. It is conceivable that students in our sample required a longer time to fully internalize mindfulness and anchor it in their daily lives. The decreases in engagement at t3 may indicate decreased identification with self-inflicted pressure resulting from performance-oriented standards.

Finally, the beneficial effects of mindfulness with respect to perceived stress, self-regulation, school-related self-efficacy and interpersonal problems among students may also be interpreted within the framework of Deci and Ryan's *Self-Determination Theory* (2000). Self-Determination Theory holds that all human beings are driven by the universal psychological needs for autonomy, competence and relatedness (Deci and Ryan, 2000). By this token, the reported effects on stress and self-regulation may have induced students in our sample to feel more self-determined and less heteronomous, thereby increasing their sense of autonomy. Moreover, it seems plausible that self-efficacious students with fewer interpersonal difficulties should believe more in their abilities to engage in learning processes and social situations, thus increasing their sense of competence and relatedness.

### Teachers

With reference to teachers, this study's results point to significant improvements among participants of the intervention group in (i) self-reported mindfulness (*d* = 0.66) and (ii) interpersonal problems (*d* = 0.49), after participation in an MBSR-course, relative to the waitlist control group. In light of the small sample size, the effect sizes for anxiety (*d* = 0.46) and emotion regulation (*d* = 0.55) moreover indicate a benefit in these areas. In general, all outcome variables, with the exception of engagement and creativity, changed in the hypothesized direction, and may well yield significant results in a larger sample.

The increase in self-reported mindfulness as measured by the FMI indicates that the implemented intervention did indeed foster central mindful attitudes, namely acceptance and presence, among participating teachers. While research on the effects of mindfulness interventions with teachers in particular remains scarce, this finding is consistent with international studies to date, whereby mindfulness interventions resulted in increased mindfulness levels among teachers (Poulin et al., 2008; Gold et al., 2010; Kemeny et al., 2012; Meiklejohn et al., 2012; Flook et al., 2013; Jennings et al., 2013; Roeser et al., 2013).

The effects on mindfulness are of two-fold relevance. Firstly, increased mindfulness has been associated with improved mental health generally (e.g., Khoury et al., 2013) and among teachers in particular (Poulin et al., 2008; Franco et al., 2010; Gold et al., 2010; Mañas et al., 2011; Kemeny et al., 2012; Flook et al., 2013; Jennings et al., 2013; Roeser et al., 2013; Weare, 2014a). Since teachers are often severely ailed by stress and at risk for burnout (Bauer et al., 2007; Unterbrink et al., 2008; Bauer, 2009), the increases in mindfulness reported here may in the long run constitute an asset to teacher mental health. While group comparisons of mental health variables in our study (stress, anxiety and depression) did not yield significant results, the medium effect size found for anxiety supports this interpretation. Similarly, within-group comparisons of pre-post scores point to a significant reduction in anxiety in the intervention group, further corroborating a promising impact on health-related variables.

Secondly, the increases in self-reported mindfulness are of particular value to the teaching profession, since mindfulness among teachers has been shown to contribute to improved teaching practices and student-teacher relations as well as a more constructive classroom climate. Studies in this area often depart from the intuitive assumption that teachers who are not severely stressed are likelier to invest more energy and devotion into their profession (Jennings and Greenberg, 2009). Further, these studies posit that mindfulness may serve to foster nurturing classroom attitudes and interactions (Flook et al., 2013). Indeed, considering mindfulness a “habit of mind,” Roeser et al. (2012) note a rise in professional development programs for teachers based on mindfulness techniques. Teachers, by virtue of the very nature of their job, are exposed to a set of tasks as challenging as they may be rewarding. By definition, these tasks largely consist of high-intensity interactions with students, colleagues, parents, and social activities that require vigilance, self-management and constantly renewed self-motivation (Keller et al., 2014; Weare, 2014a). It has been argued that this type of occupation entails high levels of “Emotional Labor” (Keller et al., 2014), which the authors associate with *surface acting* and *expressive suppression*. In other words, teachers often feel compelled to manage negative emotions such as anger, frustration or insecurity, such that they don't manifest visibly or directly shape social interactions. Whereas Emotional Labor of this kind has been associated with emotional exhaustion (which in turn is central to the burnout syndrome), mindfulness may offer an alternative approach to the complex challenges that teachers face. Rather than suppress or mask negative emotions that are bound to arise in the school setting, mindful teachers may be able to develop a different attitude to these emotions. They may recognize and accept them in a more generous and dis-identified spirit, allowing them to arise and then pass, without feeling the compulsion to act or react to them immediately.

Mindfulness and mindfulness-based interventions have indeed previously yielded auspicious results on an interpersonal/interactive level. Singh et al. (2013) for instance demonstrated decreases in difficult behaviors and negative interactions as well as an increase in compliance among preschool students after their teachers underwent 8 weeks of mindfulness training. Similarly, Jennings (2015) described a correlation between several mindfulness facets among teachers (*n* = 35) on one hand and classroom climate as well as student interactions on the other hand. More specifically, the author noted that the dimensions “awareness,” “non-judge,” and “describe” of the *Five Facets Mindfulness Questionnaire* (FFMQ, Baer et al., 2006) were associated with emotional support in the classroom (as measured by a standardized classroom observational coding system). The dimension “observe” furthermore correlated with perspective-taking toward whichever student the teacher considers the most challenging, while the dimension ‘awareness’ correlated with sensitivity of discipline, as measured by a standardized teacher interview. On a similar note, Napoli (2004) conducted in-depth interviews with teachers who participated in an 8-week mindfulness course combined with in-class mindfulness instruction sessions. The participants reported beneficial impacts of acquired mindfulness skills on classroom interactions as well as coping with situations of conflict and anxiety, among other things. In another study, educators and parents of children with special needs (*n* = 70) who took part in a randomized, controlled waitlist mindfulness-based intervention, showed significantly higher relational competences (i.e., empathic concern and forgiveness; Benn et al., 2012). Moreover, in an elaborate research study by Kemeny et al. (2012), findings indicated higher emotion recognition skills and compassion as well as lower hostile behavior (as assessed by a marital interaction task) in an experimental group that participated in a training program that incorporated mindfulness, relative to a control group.

The potential of fostering mindfulness specifically among teachers is also echoed in the significant reduction of interpersonal problems in our intervention group relative to the waitlist group. Interpersonal problems constituted the only outcome variable that was significantly affected among both students and teachers. At follow-up, this tendency was not only maintained but continued, albeit non-significantly. Fewer interpersonal problems and the likely implications thereof for teacher-student relationships are central to the rationale of our study. On one hand, the relationship to students plays a considerable role with respect to teacher mental health (Bauer et al., 2006, 2007; Bauer, 2009; Jennings and Greenberg, 2009; Schaarschmidt, 2010; Jennings, 2015). On the other hand, the relationship between students and teachers is crucial for improving the climate of the educational setting at large (Flook et al., 2013; Jennings et al., 2013). The effect on interpersonal problems is likely related to the changes in emotion regulation skills among participants of the intervention group, which yielded a medium effect size (however not at a non-significant level). Emotion regulation and interpersonal skills are closely intertwined and lie at the heart of Social and Emotional Competences (SEC) which in turn are vital for effective teaching, supportive classroom management as well as teacher-student-relationships and teacher health (Jennings and Greenberg, 2009).

With respect to the changes in the intervention group at follow-up, within-group comparisons of pre-post levels indicate largely stable outcomes for most variables. However, a non-significant increase in stress, depression and anxiety was reported compared to t2. These tendencies suggest that perhaps generally increased workload at the time of follow-up assessment may have contributed to our results. A definitive evaluation of the sustainability of the changes reported here is difficult based on these pilot data.

In sum, the reported results for teachers are of high relevance to this population in that they can be expected to bolster mental health and may contribute to improved classroom interactions and climate. As touched upon in the introduction, teachers would benefit from additional resources that tap the potential and resilience factors within this profession and that counterbalance pronounced health risks in the field. Our results support the assumption that not only does mindfulness constitute one such resource in itself; it may also assist teachers in tapping other interpersonal resources that the school setting offers.

### Teachers and students in comparison

We consider it a particular strength of our intervention that we addressed both students and teachers in an effort to impact the educational space as a whole. Comparing and interpreting the different results among teachers and students is therefore of particular interest in this context.

Overall, students exhibited significant improvements in stress, self-regulation, school-specific self-efficacy, and interpersonal problems, while solid effect sizes on mindfulness, anxiety, and creativity indicate a realistic potential in those areas. By contrast, teachers most notably showed higher mindfulness levels, fewer interpersonal problems, and promising effect sizes on emotion regulation and anxiety. These somewhat different response patterns may be due to the developmental stages of the two respective populations as well as the different life tasks and realities they are confronted with.

It stands to reason that instructing students in mindfulness may serve to modulate a crucial interaction within adolescent development, rendering young practitioners more aware of diverse influences on their motivation, goals and behavior as well as potentially enabling them to cope with unwanted or counterproductive influences. In that sense, mindfulness may contribute to strengthening adolescents' self-regulation skills, thereby increasing the likelihood of positive growth trajectories. Against this background, the reported changes in stress, self-regulation, self-efficacy, and interpersonal problems indeed suggest such a positive growth trajectory and reflect the synthesized effect of increased awareness of both external influences and intrinsic needs and goals translated into action and increased self-determination.

Teachers on the other hand are in a developmental stage that is less susceptible, particularly on a neurological level. Unlike the relatively fluid and dynamic psychological make-up of adolescents, many character traits and skills among adults have developed and solidified over the years. This often allows for only a slim margin of change and improvement and likely causes ceiling effects on many of the investigated outcome variables. Hence, while students seem to profit from the course with respect to self-efficacy and self-regulation, teachers may not reap significant benefits in those areas since, arguably, they have already acquired adequate mastery of those skills. This is all the more likely as this mastery is somewhat necessary for becoming a teacher to begin with.

Consequently, teachers may be likelier to respond to a mindfulness intervention on variables that are closely associated with their daily lives and contexts and less dependent on changes at trait level. This may account for the effects in interpersonal problems and the medium effect sizes on anxiety and emotion regulation. Measured at state level, these outcome variables are sensitive to change and reflect a more immediate impact of acquired mindfulness competences on teachers' complex personal and professional lives. Thus, it may be argued that the different response patterns of teachers and students in the present sample are due to the type of stressors and challenges that the respective population copes with. In the case of teachers, the stressors stem from a profession often associated with Emotional Labor and intense social interactions, while the reality of students is predominantly characterized by stressful performance standards, peer pressures and age-specific developmental tasks.

Interpersonal problems constitute the only variable that improved consistently across both populations, indicating a uniform effect of mindfulness in this area. As mentioned before, this improvement is particularly valuable insofar as it signifies the possibility of an overarching impact of mindfulness on educational constellations and contexts through improving teacher-student-relationships. Another factor possibly contributing to the positive effect on interpersonal problems among both populations may be the cohesion and closeness developed by virtue of the group setting of the MBSR-course. Being part of a group intervention and regularly sharing thoughts and experiences may well impact individual interpersonal approaches. Our results regarding interpersonal aspects are of particular relevance since the majority of psychological measures in mindfulness research to date are limited to dimensions of individual traits and states as well as personal well-being and behavioral regulation. Thus, the meta-analysis by Sedlmeier et al. (2012) found that only four out of 125 studies assessed interpersonal aspects of meditation, even though interpersonal variables obtained the largest effect size of all other psychological areas in this meta-analysis.

However, there are also outcome variables that were unaffected in both samples. While it is noteworthy that the sample size is an important limitation in this regard, possible explanations for the lack of effects on openness and engagement are in order. It is conceivable that openness, as a trait variable, is generally more difficult to impact and psychometrically less sensitive to change. The meta-analysis by Sedlmeier et al. (2012), for instance, reported the lowest effect size for neutral personality variables (*r* = 0.03), of which openness is an example. As for the lack of increase in engagement, it is possible that increased mindfulness can relax one's attitude toward the demands and role-specific tasks one faces rather than deepening one's commitment to and identification with one's work. This interpretation is consistent with the fact that engagement actually decreased after the intervention in the present sample, although not at a significant level. In fact, studies have shown that mindfulness can induce employees to view their job conditions more critically and to distance themselves from difficult job-related situations (Walach et al., 2007). We therefore argue that a more mindful attitude could be associated with a healthy detachment from performance pressure and outcome-oriented work styles, in favor of a more accepting and serene approach to the tasks one is posed. Nonetheless, further research determining the exact nature and implication of such a hypothesized detachment is needed.

Finally, creativity yielded a moderate effect size among students, indicating that the variable may respond in a larger sample. However, no effects were found in the teacher population. While it is generally exceedingly difficult to capture creativity and changes therein, it is possible that the task used to assess creativity was less than optimally suited for teachers. Teachers may have not taken the drawing test very seriously, especially at t2, when they were presented with the same task a second time. Students, by contrast, may be more amenable to this type of task. It may be advisable therefore to complement assessment of teacher creativity with a different instrument (for instance a verbal instrument) before drawing definitive conclusions.

### Limitations

While the present study exhibits several strengths and promising results, a number of limitations must be pointed out. First and foremost, the representativeness of the findings reported here must be viewed with caution, due to the targeted type of school (catholic) and the rather homogenous sample (all-female student population). Similarly, the relatively small sample size and hence limited statistical power necessitate an emphasis on the pilot character of this project. We maintain that focusing on the selected school within the present context was a legitimate choice not only logistically, but also in light of the higher psychological vulnerability of female adolescents and the state of research in this field in Germany. Nonetheless, further research in more generalizable settings is recommended.

Furthermore, due to the considerable time constraints within school settings, the participants of this study could not be assigned randomly to the intervention and waitlist group, which may have affected group characteristics and course impact. In our opinion, this disadvantage is at least partially outweighed by the significant drop out and non-participation of teachers and students had their schedules and preferences not been taken into account.

The extensive reliance on self-report questionnaires constitutes another limitation to the present findings, since this type of data is more susceptible to bias than more objective measures, such as physiological parameters, for instance. Replicating the results of this study with additional physiological or behavioral assessments (e.g., cortisol level or observational data) may further our understanding of the mechanisms of mindfulness interventions and validate the present findings.

Moreover, a number of confounding context variables inherently part of in-field projects may have contributed to the impact of the intervention. These include, but are not limited to: (i) the fact that the course was conducted on school premises and therefore subject to a number of organizational and logistical disruptions, (ii) the interference of stressful school periods (i.e., examinations), and (iii) the different group dynamics resulting from the relationships and constellations among teachers and students, respectively. These types of shortcomings are largely inevitable in fieldwork of any kind and especially so in the highly complex educational field; they must therefore be considered in relation to the ecological validity and relevance of the generated data.

In terms of assessment, three of the measures used in this study were not validated for adolescent age groups (FMI, ERSQ, and PSQ). Even though in our judgment all items were adequate for our target groups and purposes, the lack of psychometric properties specifically for adolescent populations constitutes a shortcoming that could be remedied in further studies if suitable alternative instruments are found.

Finally, while we did measure effects on outcome variables at a 4 month follow-up point, little can be said about the long-term sustainability of results, or the unfolding of long-term effects. This is a general issue with mindfulness research that remains to be elegantly addressed in future studies. Furthermore, if mindfulness is to impact school systems in a sustainable and structural manner, school administrations would have to take measures to this effect. These may include reminders of mindfulness practice in the daily lives of students and teachers, recurrent mindfulness-related inputs, spaces for meditation and quiet retreat on school premises as well as the potential integration of mindfulness contents in school curricula and teacher trainings.

## Concluding remark

This project aspires to reinvigorate the ancient Greek etymology of the word “school” with fresh relevance and render the experience of students and teachers in German schools more rewarding and congruent with individual well-being, priorities and values—in other words: more mindful. Overall results indicate that this is a feasible endeavor. It is however crucial in this respect to point out that mindfulness practice should not be considered a panacea for dysfunctions possibly rooted in the underlying school system, or the cure-all for no matter what individual problems. It is likewise vital to emphasize that sustainable and sound improvements to the educational field cannot and should not stem solely from individual effort, and that this research is by no means holding individuals exclusively responsible for their own wellbeing.

Especially in light of the attention that media and public discourse are currently paying to the concept of mindfulness, appraisals of this practice must remain grounded in scientific research and hitherto ascertained knowledge about what mindfulness can and cannot accomplish. The research at hand furthers this knowledge by virtue of its specific strengths: a focus on research questions largely unaddressed thus far and a dual approach that targets both students and teachers in a controlled study design.

## Author contributions

All authors listed, have made substantial, direct and intellectual contribution to the work, and approved it for publication.

### Conflict of interest statement

The authors declare that the research was conducted in the absence of any commercial or financial relationships that could be construed as a potential conflict of interest.

## Footnotes

^1^The project at hand is part of a larger, interdisciplinary Collaborative Research Centre titled “Muße. Concepts, Spaces, Figures.” The German word Muße corresponds to the Latin equivalent *otium* and the ancient Greek *scholé*; there is no exact English translation. In theory, Muße may be characterized as moments of fulfillment and serenity when time perception is suspended. In Muße, one is free to engage in whatever activity one chooses with immersion, satisfaction and gratuitous dedication. Across 15 projects spanning a number of diverse disciplines, this CRC is largely interested in the historical, philological, philosophical, theological, ethnological, sociological, and psychological dimensions of Muße and the conceptual implications and relevance of cultural debate around it.

^2^The project was in contact with this specific school as a result of previous professional cooperation. Based on the gender differences in internalizing symptoms discussed above, an all-girl student population was deemed justified within the context of a pilot study.
